# UPLC/MS^n^ analysis of *Bougainvillea glabra* leaves and investigation of antioxidant activities and enzyme inhibitory properties

**DOI:** 10.1038/s41598-025-11851-9

**Published:** 2025-08-02

**Authors:** Heba A. S. El-Nashar, Omayma A. Eldahshan, Mahmoud A. El Hassab, Gokhan Zengin, Esraa A. Elhawary

**Affiliations:** 1 Department of Pharmacognosy, Faculty of Pharmacy, Ain Shams University, Abbassia, Cairo, 11566 Egypt; 2 Department of Medicinal Chemistry, Faculty of Pharmacy, King Salman International University (KSIU), South Sinai, Egypt; 3 Deparment of Biology, Science Faculty, Selcuk University, Konya, 4210 Turkey

**Keywords:** *Bougainvillea glabra*, Nyctaginaceae, Metabolites, UPLC/MS^n^, Antioxidant, Enzyme Inhibition, Plant sciences, Medical research

## Abstract

*Bougainvillea glabra* is a well-known and well-documented ornamental plant belonging to family Nyctaginaceae, always planted for its beautifully colored flowers. The current study was performed for profiling the metabolites of *B. glabra* leaf extract. Further, the leaf extract was evaluated using the total phenolic and total flavonoid assays and profiling using UPLC/MS^n^ for the leaf extract’s secondary metabolites. The leaf extract was also subjected to several antioxidant assays, viz. DPPH, ABTS, CUPRAC, FRAP, metal chelating, and phosphomolybdenum tests, as well as enzyme inhibition assays such as α-amylase, α-glucosidase, acetylcholinesterase (AChE), and butyrylcholinesterase (BChE). The results showed that the total phenolic and flavonoid contents were 27.68 mg GAE/g and 31.76 mg RE/g, respectively. Besides, twenty-one metabolites were tentatively identified and quantified, where flavonoids and phenolic acids constituted the most abundant classes of compounds. The molecular docking experimentss showed that the most abundant components, namely rhamnocitrin-*O*-rutinoside, sagerinic acid, tri-*O*-caffeoyl-shikimic acid, and chlorogenic acid, had the best scores when docked in the vicinity of the selected enzyme targets. The extract recorded a potentially powerful antioxidant activity, compared to the used standards, while it showed a good inhibitory effect against AChE (2.40 mg GALAE/g) and BChE (1.95 mg GALAE/g). The tyrosinase-inhibiting effect was 48.23 mg CAE/g. The amylase and glucosidase inhibitory effects were 0.30 mmol ACAE/g and 0.03 mmol ACAE/g, respectively. Thus, this study suggests that *B. glabra* may not only act as an ornamental plant, but also it may be a promising source for effective phytochemicals that act as antioxidants and enzyme inhibitors, which may play a role in reversing the aging process and age-related ailments like diabetes.

**Supplementary Information:**

The online version contains supplementary material available at 10.1038/s41598-025-11851-9.

## Introduction

Medicinal plants are a treasure trove of nature’s healing power, deeply rooted in human history and traditional medicine systems. From ancient herbal remedies to modern pharmaceutical discoveries, these plants play a significant role in healthcare and scientific research^1–3^. Plants have been a crucial component of human life and society, serving our basic needs for food, medicine, and shelter, but their importance goes beyond these necessities^4,5^. Plants play a critical role in human health and well-being by providing essential nutrients, such as vitamins, minerals, and fiber, which are critical for proper bodily function^6,7^. By incorporating a variety of plant-based foods into our diets, we can improve our overall health and reduce our risk of chronic diseases such as heart disease, diabetes, and cancer^8,9^.

Genus *Bougainvillea* from the family Nyctaginaceae is one of the widespread genera around the world. It comprises eighteen species and is native to South America. This genus is popular for its members, which possess flowers that bloom throughout the year in different colors according to the species, cultivars, or hybrids^10^. The anthocyanins from *Bougainvillea* flowers are widely applied as a natural source for pigments in cosmetic, textile, and pharmaceutical industries, providing higher safety than synthetic coloring agents^11,12^. In folk medicine, *B. glabra* was used for alleviating asthma, cough, pertussis, bronchitis, ulcers, microbial infections, and diarrhea. In Panama, flowers were used for hypotension, while in India, different parts were utilized to treat many ailments, such as stomach acidity, hepatitis, diarrhea, cough, blood vessel problems, and sore throat. In addition to that, the decoctions of *B. glabra* were used in Nigeria to mitigate many cases of inflammation, intestinal disorders, and pain^13–15^. *B. spectabilis* was reported traditionally to alleviate ulcers, pain, and inflammation^16^ as well as its use in treating respiratory system-related diseases such as pertussis, cough, flu, lung pain, bronchitis, and snoring^13^. Upon reviewing literature on *B. glabra*, the plant was reported to have antimicrobial, antioxidant^16^, anthelmintic, antidiabetic, anticancer, antidiarrheal, antihyperlipidemic^17^, cardioprotective, neuroprotective, antifertility^18^, antiulcer^19,20^, anti-inflammatory^21^, antibacterial^22^, anti-urolithic, and analgesic activities^14,15^. Phytochemically, the genus *Bougainvillea* is rich in aliphatic hydrocarbons, fatty acids, fatty alcohols, phenolic compounds, flavonoids, volatile compounds, peltogynoids, phytosterols, terpenes, carbohydrates, and betalains^10^.

Reactive oxygen species/reactive nitrogen species are the most common terms used to define chemically active molecules containing oxygen, nitrogen, or both that react with other molecules in cells^23,24^. Their production has gained biological importance not only for their role in signaling pathways but also for their contribution to tissue damage and disease when at high levels^25^. ROS/RNS levels within the physiological range are critical for many redox-dependent signaling processes, such as cellular adaptation to stress, inflammation, or aging, as reviewed previously^26,27^. However, since the production of ROS is described to increase with age, the role of these reactive species becomes even more relevant in the pathophysiology of age-related diseases^28^. Diabetes *mellitus*, especially type 2 (T2DM), is a global health concern, particularly in the elderly group^27^. Along with an increased number of senescent pancreatic *β*-cells and obesity, aging is considered a major risk factor for T2DM^29^. Regarding ND, particularly Alzheimer’s and Parkinson’s disease, both are considered progressive neurological disorders, caused by the neurodegeneration of nervous cells in specific brain regions and with a great incidence in older populations^30^. Cancer is also considered an age-related disease, as there is a 20% risk of developing cancer before 75 years^31,32^.

This study was developed in order to assess the total phenolic and flavonoid content of 80% methanol extract of *B. glabra* followed by qualitative and quantitative identification of its main metabolites. Moreover, an analysis of the potential antioxidant and enzyme inhibitory activity of such an extract.

## Materials and methods

### Plant collection and extract Preparation

The fresh leaves of *B. glabra* were obtained from El-Zohraya Garden, Giza, Egypt, in February 2023. The leaves and fruits were kindly provided by Mrs. Therese Labib, Plant Taxonomy Consultant at the Ministry of Agriculture and El-Orman Botanical Garden, Giza, Egypt. The collection of plant material was established in compliance with the national guidelines. A voucher specimen (Number: PHG-P-PG-523) is deposited at the Department of Pharmacognosy, Faculty of Pharmacy, Ain Shams University, Cairo, Egypt.

The plant leaves (500 g) were chopped into small pieces and extracted with 80% methanol (6 L) and repeated three times till exhaustion^33^. The collected extracts were evaporated using BUCHI Rotavapor^®^ R-300 under reduced pressure at 45 °C until complete dryness, yielding 16.96 g of brown material. Then, the obtained amount was lyophilized and stored at -4 °C in refrigerator till biological investigation.

### UPLC/MS^n^ analysis conditions

The phytochemical analysis of *B. glabra* extracts was assessed according to the previously reported method using high-performance liquid chromatographic (HPLC) analysis joined with an ESI-MS/MS spectrometer detector^34^. This technique allowed tentative identification of phytoconstituents based on the molecular weights. The plant extract (100 µg/ml) was dissolved in methanol (HPLC-grade), then filtered *via* a membrane disc (0.20 μm). Then, the filtrate (10 µL) was injected into HPLC-ESI-MS/MS. The used HPLC instrument has the following specifications: Waters^®^ stocked with a reversed-phase C-18 column (ACQUITY UPLC-BEH C-18, particle size ~ 1.7 μm, dimensions = 2.1 × 50 mm). Before injection, the mobile phase was filtered through a membrane disc filter (0.2 μm) and sonicated. The elution run took 35 min using gradient elution (water and methanol acidified with 0.1% formic acid) with a flow rate of 0.2 mL/min. On an XEVO TQD triple quadrupole instrument, positive and negative ions were acquired using ESI-MS. Waters^®^ Corporation, Milford, MA 01757, U.S.A. supplied the HPLC unit and mass spectrometer. Edwards^®^, U.S.A., provided the vacuum pump at desolvation temperatures of 150 and 440 °C. The mass spectra were obtained using the software Masslynx 4.1 at an ESI range *m/z* of 100–1000. To tentatively identify the obtained mass spectra, the peak retention time (tR) and their fragmentation pattern were compared with the reported data in the literature.

### Assay for total phenolic and flavonoid contents

Total phenolics and flavonoids were quantified according to the procedures outlined by^35^. Gallic acid (GA) and rutin (R) were used as reference standards in the studies, with results expressed as gallic acid equivalents (GAE) and rutin equivalents (RE).

### Molecular docking studies

The X-ray 3D structures of butyrylcholinesterase, acetylcholinesterase, α-amylase, and α-glucosidase were downloaded from the protein data bank using the following IDs: 6esj, 1f8u, 4gqq and 3wy2, respectively. Vina autodock and MGL tools were employed to conduct docking studies^36,37^. The four major compounds identified in the extract of *B. glabra* leaves were implemented in the docking study. All four receptors and the four compounds were saved in a pdbqt format using MGL tools as an essential requisite by Vina Autodock. The active site of each target was determined from the binding of the corresponding co-crystallized ligand. Finally, the docking results were inspected by Discovery Studio Visualizer, which was also used to generate the 2D interaction diagrams.

### Assays for *in vitro* antioxidant capacity

By the methodologies detailed in our prior publication^38^, various antioxidant tests were carried out. The outcomes were represented as milligrams of trolox equivalents (TE) per gram for the DPPH, ABTS radical scavenging, CUPRAC, and FRAP tests. In millimoles of TE per gram of extract, the phosphomolybdenum (PBD) test examined antioxidant potential, and in milligrams of disodium edetate equivalents (EDTAE) per gram of extract, the metal chelating activity (MCA) was determined.

### Inhibitory effects against some key enzymes

In accordance with the established protocols^38^, experiments on enzyme inhibition were performed on the samples. Acarbose equivalents (ACAE) per gram of extract were used to measure the activities that inhibit amylase and glucosidase, while milligrams of galanthamine equivalents (GALAE) per gram of extract were used to examine the inhibition of acetylcholinesterase (AChE) and butyrylcholinesterase (BChE). The amount of tyrosinase inhibition for each gram of extract was measured in milligrams of kojic acid equivalents (KAE).

## Results and discussion

### Plant metabolites identified through UPLC/MS^n^ of *B. glabra* leaf extract

**Fig. 1:**
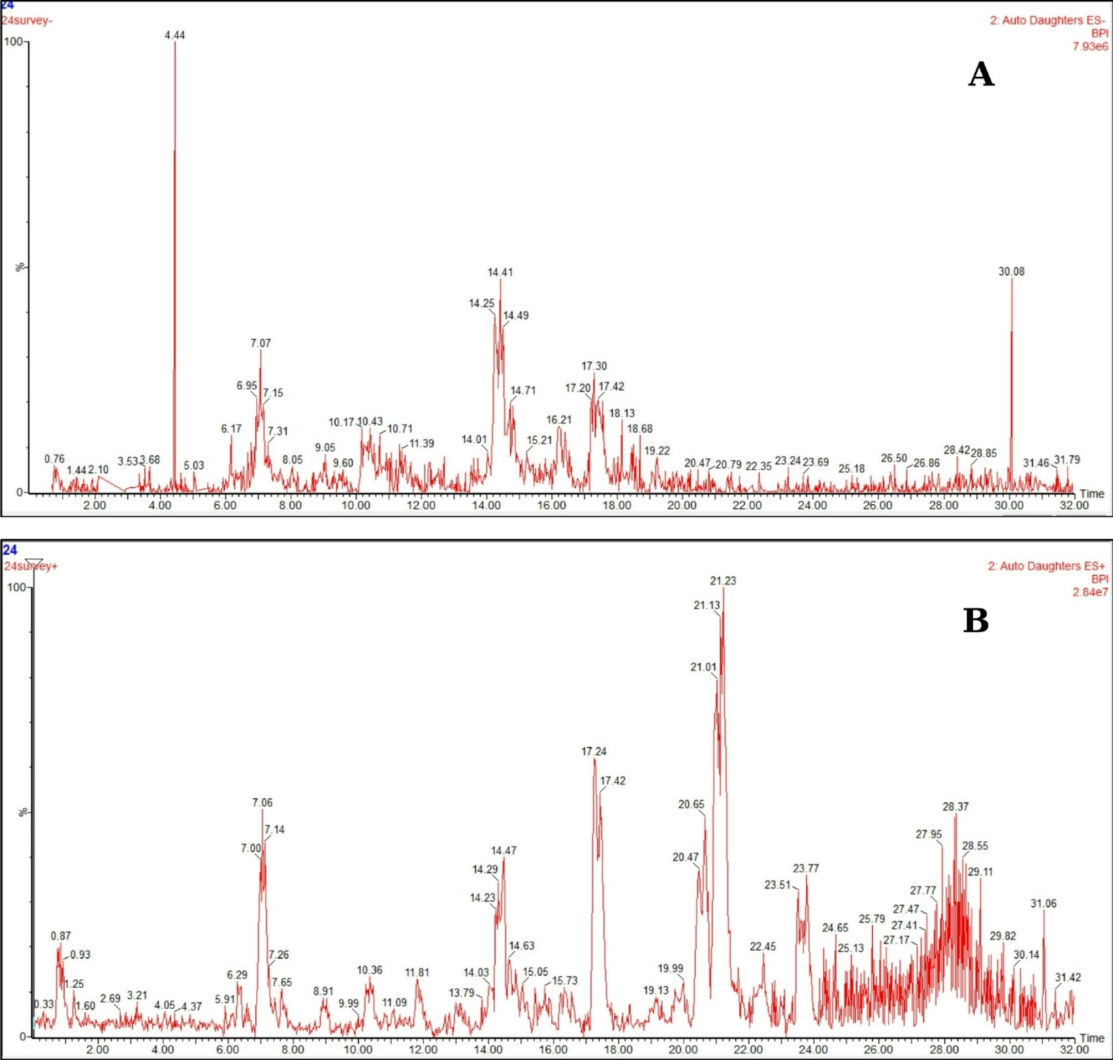
The UPLC/MS^n^ BPI chromatograms in (**A**) negative ion mode and (**B**) positive ion mode.

The methanol leaf extract of *B. glabra* was analyzed using ultra-performance liquid chromatography coupled with tandem mass spectrometry (UPLC/MS^n^) to identify its main phytoconstituents. Negative and positive ionization techniques were applied in order to detect different classes of metabolites (Fig. 1A and B). The % identification was (93.57%, -ve mode and 39.70%, +ve mode). Twenty-three peaks were detected, from which two peaks were marked as unidentified, and the remaining twenty-one compounds were tentatively identified and recorded in Table 1. The identified components were listed below according to their chemical class.

**Table 1:** Binding scores for the tentatively identified components against the five targets.

	6esj Butyrylcholinesterase	1f8u Acetylcholinesterase	4gqq α-Amylase	3wy2 α-Glucosidase
Rhamnocitrin-*O*-rutinoside	− 17.2	− 14.8	− 9.8	− 16.9
Sagerinic acid	− 11.5	− 12.9	− 9.7	− 11.6
Tri-*O*-Caffeoyl shikimic acid	− 10.7	− 9.2	− 7.3	− 12.9
Chlorogenic acid	− 12.5	− 9.4	− 6.4	− 10.4
Reference compound	− 13.7	− 7.8	− 7.8	− 15.2

#### Flavonoids

Six flavonoids and two biflavonoids were detected (Table S1). A deprotonated peak was found at *m/z* 593 with fragments at *m/z* 501, 372, 285, and 236. The fragment at *m/z* 285 is due to the luteolin skeleton; thus, this compound was assigned to luteolin robinobioside^39^. Similarly, another peak was detected at *m/z* 609 (+ ve mode, 13.60%) with fragments at *m/z* 579, 525, 463, 393, 371, 311, and 281, and it was tentatively identified as rhamnocitrin-*O*-rutinoside^40^. Compounds 6,7, 8, and 21 presented parent peaks at *m/z* 563, 315, 497 and 581, respectively with their fragments detected at (*m/z* 501, 395, 359, 343, 303 & 191), (*m/z* 300, 176 & 65), (*m/z* 461, 437, 327, 299, 269 & 183) and (*m/z* 565, 525, 481, 462, 453, 394, 356, 329, 261, 245, 179), respectively which lead to their identification as apigenin-*C*-hexoside-*C*-pentoside^41,42^, isorhamnetin^39^ and brevifolin-di-carboxylic acid hexoside^43^ and di-hydro-heveaflavone^44^, respectively. In addition, two peaks were detected for biflavonoids namely; di-hydro-bi-apigenin methyl ether (*m/z* 553 with fragments at *m/z* 535, 471, 441, 353, 331, 311, 209)^44^ and manniflavanone (*m/z* 589 –ve, *m/z* 605 + ve with fragments at *m/z* 521, 453, 443, 385, 317, 249, 181)^45^.

#### Phenolic acids

Phenolic acids marked the second most abundant class after flavonoids, with five phenolic acid derivatives detected in Table 1. One parent peak was shown at *m/z* 341 with one fragment at *m/z* 131; thus, it was tentatively defined as caffeic acid hexoside^39,46^. Two other caffeic acid derivatives were tentatively identified as tri-*O*-caffeoyl shikimic acid^47^ and chlorogenic acid^48^ with parent peaks at *m/z* 659 (31.39%) and *m/z* 353 (+ ve mode, 25.00%), respectively and daughter peaks at (*m/z* 571, 475, 397, 329, 299 & 285) and (*m/z* 331, 285, 317 & 203), respectively. Moreover, two deprotonated molecular ion peaks were found at *m/z* 359 and *m/z* 521, which were defined as rosmarinic acid^42,49^ and its derivative, rosmarinic acid hexoside (3.52%)^48,50,51^, respectively.

### Lignin

One peak was detected for a lignin derivative, which was previously identified from the same genus, this peak was found at *m/z* 579 with MS/MS at *m/z* 521, 485, 475, 431, 389, 379, 343, 315, 299, and 283 then it was tentatively identified as (+)-syringaresinol hexoside which is also known as acanthoside B (Table S1)^14^.

#### Disaccharide

A peak was found at *m/z* 487, and its fragments were at *m/z* 445, 427, 359, 345, 329, 301, 279, and 179 (Table S1), and it was defined as rhamnosyl sophoroside (previously identified from the same genus)^39^.

#### Anthocyanidin

A deprotonated peak was defined as delphinidin hexosyl pentosyl malonate, which showed its parent peak at *m/z* 683 in positive mode and had fragments at *m/z* 650, 562, 524, 369, 353, 331, and 303^52,53^ (Table S1).

#### Tannin

Compound 16 presented a parent peak at *m/z* 471 with fragments at *m/z* 461, 412, 359, 345, 333, 311, 309, 301, 265, and 197 (Table 1), which led to its identification as 3-methyl-epigallocatechin gallate^54^.

#### Iridoid

A deprotonated peak was detected at *m/z* 685 (6.61%) with MS/MS at *m/z* 555, 540, 471, 359, 353, 331, 309 and 265 thus it belonged to *O*-[hexosyl-caffeoyl] catalpol which is commonly known as speedoside (Table S1)^48,51,55,56^.

#### Chromene

One peak was traced at *m/z* 453(455) with its fragments at *m/z* 447, 441, 397, 361, 337, 325, 289, 265, and 191, which was then defined as *tri*-hydroxy-*di*-hydrocyclo-*penta*[b]chromene-dione-carboxylic acid hexoside (Table 1)^57^.

#### Triterpene

Oleanolic acid was tentatively identified through its deprotonated molecular ion peak at *m/z* 455 and characteristic fragmentation pattern at *m/z* 453, 441, 359, 341, 337, 329, 299, 293, 188, and 179 as shown in Table S1^58,59^.

### Determination of the total phenolic and total flavonoid contents

The total contents of phenolics and flavonoids in the tested methanol extract were determined by colorimetric methods. The results showed that the total phenolic and flavonoid contents were detected as 27.68 mg GAE/g and 31.76 mg RE/g, respectively.

### Molecular docking studies

The major compounds identified in the extract of *B. glara* leaves namely,  rhamnocitrin-*O*-rutinoside, sagerinic acid, tri-*O*-caffeoyl-shikimic acid, and chlorogenic acid, were docked into the active site vicinity of the four enzymes (i.e., butyrylcholinesterase, acetylcholinesterase, *α*-amylase, and *α*-glucosidase). As presented in Table 1, all compounds achieved acceptable binding scores when docked with the five targets.

**Fig. 2:**
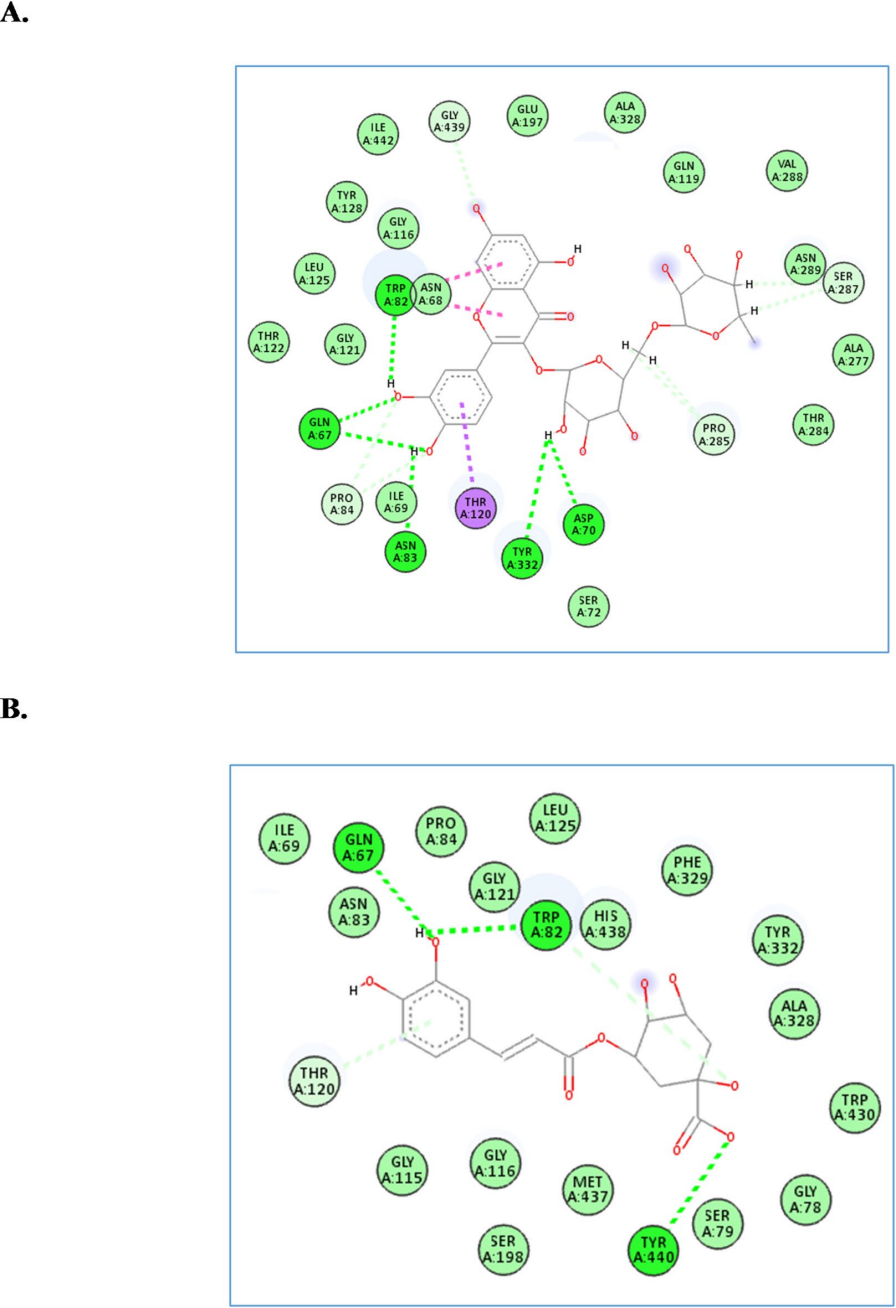
The molecular docking studies of rhamnocitrin-*O*-rutinoside (**A**) and chlorogenic acid (**B**) in the active site of butyrylcholinesterase enzyme (PDB code: 6esj).

For the Butyrylcholinesterase, the four compounds achieved docking scores from − 10.7 to -17.2 Kcal/mol, where rhamnocitrin-*O*-rutinoside and chlorogenic acid were the best compounds, achieving scores of -17.2 and − 10.7 Kcal/mol, respectively. Inspecting Fig. 2, rhamnocitrin-*O*-rutinoside interacted with Gln67, Asp70, Trp82, Asn83 and Tyr332 through hydrogen bond interactions and with, Trp82, Thr120, Pro285 and Ser287 through hydrophobic interactions, while chlorogenic acid formed hydrogen bond interactions with Gln67, Trp82 and Tyr440 through hydrogen bond interactions and with, Trp82 and Thr120 through hydrophobic interactions.

**Fig. 3:**
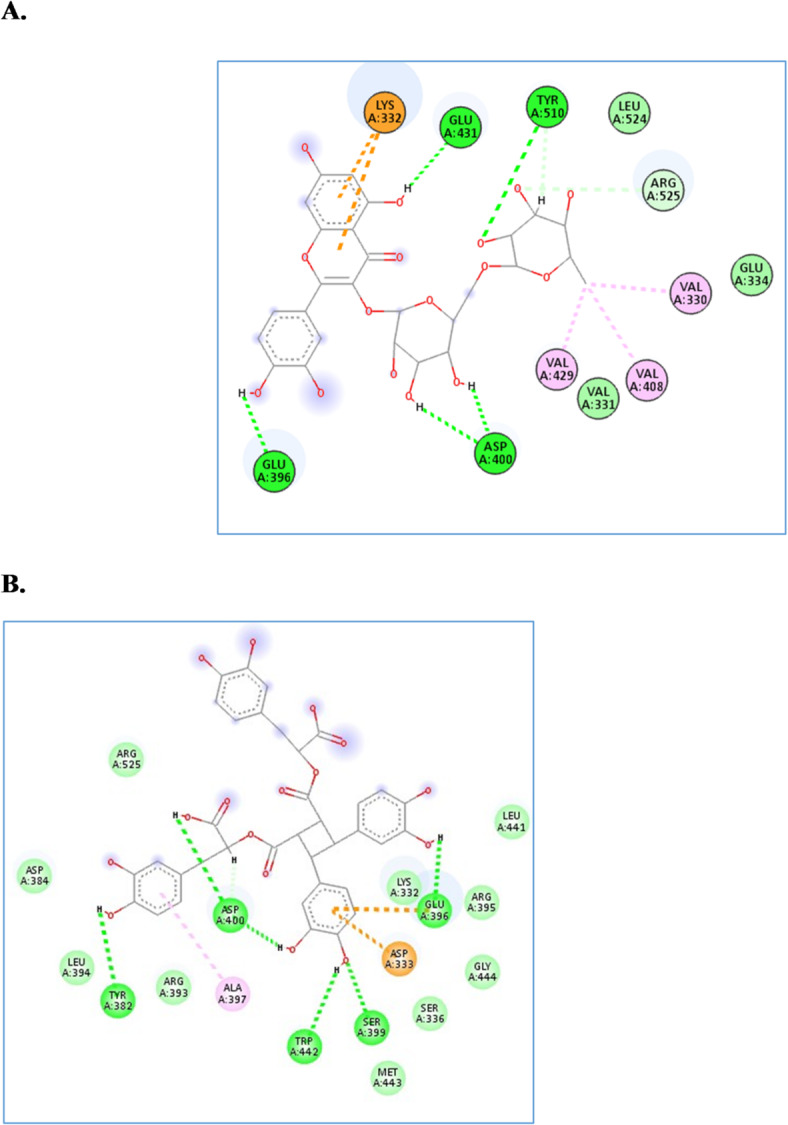
The molecular docking studies of rhamnocitrin-*O*-rutinoside  (**A**) and sagerinic acid (**B**) in the active site of AChE enzyme (PDB code: 18fu).

For the ACHE enzyme, the four compounds achieved docking scores from − 9.2 to -14.8 Kcal/mol, where rhamnocitrin-*O*-rutinoside and sagerinic acid were the best compounds, achieving scores of -14.8 and − 12.9 Kcal/mol, respectively. As seen in Fig. 3, rhamnocitrin-*O*-rutinoside formed several hydrogen bond interactions with Glu396, Asp400, Glu431, and Tyr510, in addition to several hydrophobic interactions with Lys332, Val330, Val408, and Val429. Similarly, sagerinic acid formed hydrophobic interactions with Asp333 and Ala397 and several hydrogen bonds with Tyr382, Glu396, Ser399, Asp400, and Trp442.

**Fig. 4:**
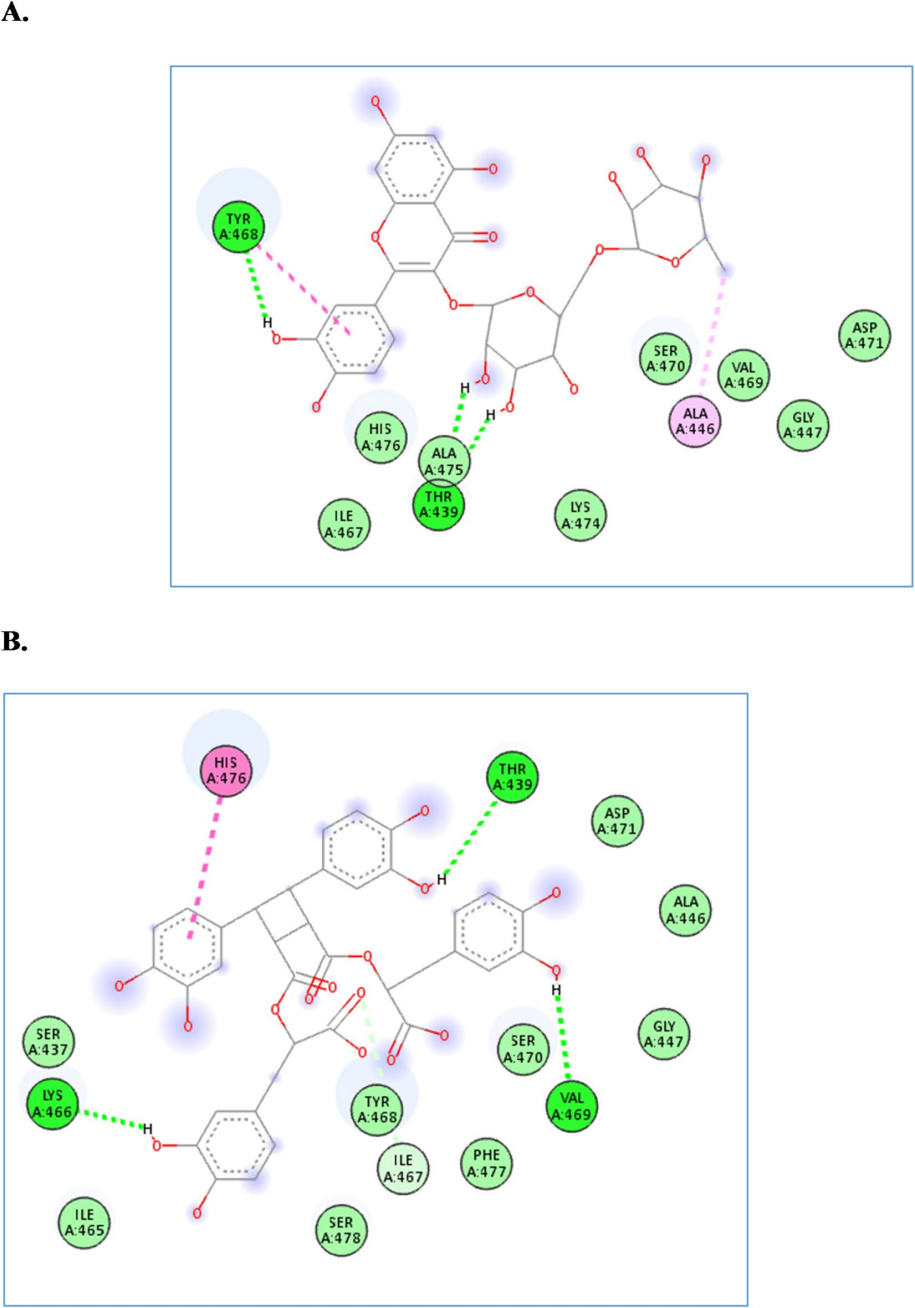
The molecular docking studies of rhamnocitrin-*O*-rutinoside (**A**) and sagerinic acid (**B**) in the active site of α-amylase enzyme (PDB code: 4gqq).

The four compounds achieved docking scores ranging from − 6.4 to -9.8 Kcal/mol against the *α*-amylase enzyme. Rhamnocitrin-*O*-rutinoside and sagerinic acid achieved the best scores, -9.8 and − 9.7 Kcal/mol, respectively. As shown in Fig. 4, rhamnocitrin-*O*-rutinoside interacted with Thr439 and Tyr468 through hydrogen bond interactions and with Ala446 and Tyr468 through hydrophobic interactions. Likewise, sagerinic acid interacted with Thr439, Lys466, and Val469 through hydrogen bond interaction and one hydrophobic interaction with His476.

**Fig. 5:**
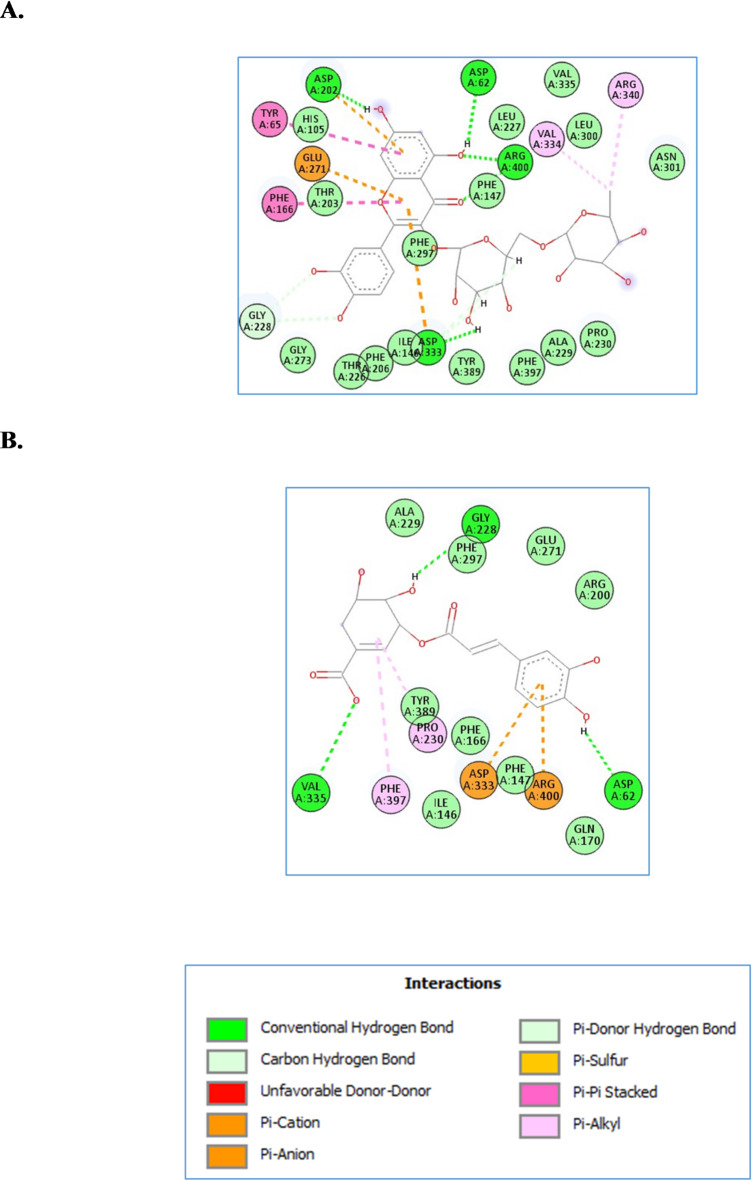
The molecular docking studies of Lupeol  (**A**) and Friedelan-3-one (**B**) in the active site of α-glucosidase enzyme (PDB code: 3wy2).

For glucosidase, the four compounds achieved excellent docking scores from − 10.4 to -16.9 Kcal/mol. Rhamnocitrin-*O*-rutinoside and *tri*-*O*-caffeoyl-shikimic acid achieved docking scores of -16.9 and − 12.9 Kcal/mol, respectively, ranking the best two compounds. Inspecting their interactions as shown in Fig. 5, it was found that rhamnocitrin-*O*-rutinoside interacted with, Asp62, Asp202, Asp333 and Arg400 through hydrogen bond interactions, in addition to several hydrophobic interactions with Tyr65, Phe166, Asp202, Glu271, Asp333, Val334 and Arg340. Similarly, tri-*O*-caffeoyl-shikimic acid interacted through hydrogen bond with Asp62, Gly228 and Val335, besides several hydrophobic interactions with Pro230, Asp333, Phe397 and Arg400.

### Antioxidant properties

The antioxidant capacity of the tested extract was examined using various chemical tests, including DPPH, ABTS, CUPRAC, FRAP, metal chelate, and phosphomolybdenum tests. The ABTS and DPPH tests were used to evaluate the radical scavenging ability, and the extract showed radical scavenging ability of 87.67 mg TE/g and 54.67 mg TE/g, respectively. CUPRAC and FRAP assays were tested to determine the reducing ability of the tested extract. In these tests, the reducing abilities were 104.41 mg TE/g and 72.75 mg TE/g for CUPRAC and FRAP, respectively. The ability in the phosphomolybdenum assay was 2.05 mmol TE/g. The metal chelating ability was 25.43 mg EDTAE/g (Table 2).

**Table 2:** Antioxidant potential of *B. glabra* leaf methanol extract.

Assay	Results
DPPH (mg TE/g)	54.67 ± 2.35
ABTS (mg TE/g)	87.67 ± 1.40
CUPRAC (mg TE/g)	104.41 ± 1.93
FRAP (mg TE/g)	72.75 ± 0.86
MCA (mg EDTAE/g)	25.43 ± 0.18
PM (mmol TE/g)	2.05 ± 0.03

Values expressed as means ± S.D. of three parallel measurements.

*GAE* Gallic acid equivalent, *RE* Rutin equivalent, *TE* Trolox equivalent, *EDTAE* Ethylenediaminetetraacetic acid equivalent.

### Enzyme inhibitory effects

The enzyme inhibitory ability of the tested extract was examined against cholinesterase, tyrosinase, α-amylase, and α-glucosidase as shown in Table 3. The extract showed a good inhibitory effect against AChE (2.40 mg GALAE/g) and BChE (1.95 mg GALAE/g). The tyrosinase-inhibiting effect was 48.23 mg CAE/g. The α-amylase and  α-glucosidase inhibitory effects were 0.30 mmol ACAE/g and 0.03 mmol ACAE/g, respectively.

**Table 3:** Enzyme inhibitory effects *of B. glabra* leaf methanol extract.

Assay	Results
AChE inhibition (mg GALAE/g)	2.40 ± 0.19
BChE inhibition (mg GALAE/g)	1.95 ± 0.25
Tyrosinase inhibition (mg KAE/g)	48.23 ± 1.45
*α*-Amylase inhibition (mmol ACAE/g)	0.30 ± 0.02
*α*-Glucosidase inhibition (mmol ACAE/g)	0.03 ± 0.01

Values expressed as means ± S.D. of three parallel measurements.

*GALAE* Galanthamine equivalent, *KAE* Kojic acid equivalent, *ACAE* Acarbose equivalent.

Genus *Bougainvillea* is considered to be one of the natural and safe sources of phytoconstituents and other beneficial components like pigments with particular use in food, textiles, cosmetics, and pharmaceutical industries. In this study, the methanol leaf extract of *B. glabra* was metabolically evaluated with the aid of UPLC/MS^n^ where twenty-one components were traced and identified as detailed in the results section. Flavonoids, phenolic acids, lignin, tannins and iridoids were among the main identified phytochemical components. The main identified components were rhamnocitrin-*O*-rutinoside, sagerinic acid, tri-*O*-caffeoyl-shikimic acid and chlorogenic acid. Upon literature review, one study evaluated the phytoconstituents of two species from the genus *Bougainvillea* namely, *B. spectabilis* Willd and *B. glabra* Choisy. The leaf and flower extracts were prepared and using UPLC/MS^n^ analysis, forty-four compounds were tentatively detected, where the major components belonged to flavonoids, anthocyanins, mono, di, triterpenes, and phenolic acids. In addition, the extracts showed potent anti-inflammatory activity comparable to piroxicam^39^. In another study, the dichloromethane (DCM) and methanol extracts were prepared from the aerial parts and flowers of *B. glabra* (Choisy). Nine major polyphenolics were identified, where the flower methanol extract was rich with catechin (6.31 µg/g), gallic acid (2.39 µg/g), and rutin (1.26 µg/g). The DCM extracts showed twenty-seven components mainly from terpenoids, alkaloids, and phenolic derivatives. All the tested extracts had intermediate enzyme inhibitory activity against tyrosinase and *α*-amylase enzymes^14^. One study examined the major compounds found in the leaves’ methanol extract of Iraqi cultivated *Bougainvillea spectabilis*. Then, the leaf methanol extract was analyzed by UPLC/MS^n^. Flavonoid glycosides such as kaempferol-3-*O*-glucoside, kaempferol-3-*O*-rutinoside, quercetin-3-rhamnoside-7-rhamnoside and quercetin-3-*O*-neohesperidoside were the major compounds^60^. In another study, the ethyl acetate fraction of *Bougainvillea* ‘Scarlett O’Hara’ cultivated in Egypt was investigated using UPLC-ESI-MS/MS, identifying fifty-seven compounds. The identified phytochemicals were seven organic acids, fourteen phenolic compounds, one betacyanin, seven anthocyanins, ten flavonoids, three saponins, six tannins, four cyclic tetrapyrolic derivatives, and five miscellaneous^52^.

Phenolic compounds are considered a great treasure with significant biological effects, including antioxidants and antimicrobial activity. Therefore, most of them have been used to produce health-promoting applications, nutrients, and medicines^61^. With this in mind, we determined the total content of phenols and flavonoids in the tested extracts. In the literature, several researchers reported different total phenolic and flavonoid contents in *B. glabra*. For example, Riaz et al.^62^ found that the total content of phenolics and flavonoids in the methanolic extract of *B. glabra* was 158.57 mg GAE/100 g of extract and 29.41 mg QE/g, respectively. The total phenolic content was also reported by Kuhn et al.^63^ as 44.98 mg GAE/g extract. Saleem et al.^64^ reported that the methanol extract of *B. glabra* contained more total phenolics (24.01 mg GAE/g) and flavonoids (41.51 mg RE/g) than the dichloromethane extract. The observed differences could be explained by geographical (altitude, soil composition, etc.) and climatic conditions (annual rainfall, sunlight duration, etc.).

The term “antioxidant” is probably one of the most popular terms in everyday life. Most people are aware of the benefits of antioxidants, which play a role in fighting free radicals. In this sense, we aimed to detect the antioxidant properties of *B. glabra*. As can be seen in Table 2, the extract possessed significant antioxidant properties in various chemical assays. In the literature, the antioxidant ability of *B. glabra* was reported by several authors. For example, Saleem et al.^64^ reported that the ABTS scavenging ability for the methanol extract of *B. glabra* was 111.29 mg TE/g. In their study, FRAP and CUPRAC values were found to be 52.13 mg TE/g and 130.41 mg TE/g, respectively. In another study by Mostafa et al.^65^, the DPPH radical scavenging ability of the acetone extract of *B. glabra* was 92.6% at a concentration of 1000 µg/ml. Saleem et al.^15^ reported that the metal chelating ability of the dichloromethane extract of *B. glabra* flowers was 17.51 mg EDTAE/g, which was lower than our current study (25.43 mg EDTAE/g). Based on Table 1, the observed antioxidant effects can be attributed to the presence of some compounds, including tri-*O*-caffeoyl shikimic, sagerinic, and chlorogenic acids. The compounds have been reported as significant antioxidants in previous studies^66–68^. For example, sagerinic acid contains multiple hydroxyl groups, and thus, due to their presence, it can effectively play antioxidant roles, including electron-donating or hydrogen-donating abilities (Lu and Foo, 2001). The ortho-trihydroxy arrangement found in gallic acid, which is part of the structure of sagerinic acid, is particularly effective for chelating metals. In addition, tri-*O*-caffeoyl shikimic contains the esterification that occurred at position 6 of shikimic acid and it can enhance its radical scavenging ability (Zeng et al., 2012).

Enzymes are the main therapeutic players in pharmaceutical applications, and most treatment strategies are based on the management of enzymes. Inhibiting key enzymes can help relieve symptoms of some diseases, such as diabetes, obesity, or Alzheimer’s disease. For example, inhibiting amylase and glucosidase can control blood sugar levels in diabetics following a high-carbohydrate diet. For this reason, several compounds (acarbose, galanthamine, kojic acid, etc.) have been synthesized as enzyme inhibitors, but most of them have unfavorable side effects. Therefore, we need to find safe and effective inhibitors instead of synthetic ones. We tested the inhibitory effect of the tested extract on clinically important enzymes. In the literature, some researchers reported the enzyme-inhibitory properties of *B. glabra*. For example, Saleem et al.^69^ reported that the amylase and tyrosinase inhibitory effects of *B. glabra* extracts were 0.09–0.12 mmol ACAE/g and 25.47–27.12 mg KAE/g, respectively, which were lower than the results of the current study. In another study^64^, the dichloromethane extract of *B. glabra* was more active on glucosidase than the methanol extract. The methanol extract (80%) of *B. glabra* exhibited an inhibitory effect on glucosidase at 37.30% at 4 mg/ml concentration, as reported by Kaisoon et al.^70^. In the relationship between structure and ability, in Table 1, some identified components, including shikimic acid, rosmarinic acid, chlorogenic acid, or rhamnocitrin, can be attributed to the observed enzyme inhibitory effects, and the compounds have been described as enzyme inhibitory agents^71–77^. For example, in a previous study conducted by Liu et al. (2020), four phenolic compounds (chlorogenic, cryptochlorogenic, neochlorogenic and caffeic acids) were investigated for tyrosinase inhibitory effects and among them chlorogenic acid exhibited the greatest inhibitory effects. Chlorogenic acid binds to copper in the active site of tyrosinase and its quinic group forms four hydrogen bonds in the peripheral site. In another study by Oboh et al. (2013), chlorogenic acid exhibited significant AChE and BChE inhibitory effects, and the presence of the OH group in its structure can be attributed to these inhibitory effects.

## Conclusion

The metabolic profiling of the methanol leaf extract of *B. glabra* led to the tentative identification and quantification of twenty-one metabolites, where flavonoids and phenolic acids, lignin, tannins and iridoids represented the most abundant phytochemicals. The extract showed significant antioxidant activity using different assays. Moreover, the enzyme inhibitory power of the extract was revealed in different enzyme assays. It could be concluded that the extract’s notable potent antioxidant and enzyme inhibitory activity could be linked to its richness with flavonoids and phenolic compounds, making it a potential source for new drug leads against many diseases such as diabetes mellitus and age-related illnesses.

## Electronic supplementary material

Below is the link to the electronic supplementary material.

Supplementary Material 1

## Data Availability

Data are available upon request from the first author. heba_pharma@pharma.asu.edu.eg.
