# Supplementary material for: UPLC/MSn analysis of Bougainvillea glabra leaves and investigation of antioxidant activities and enzyme inhibitory properties
Source: Sci Rep. 2025 Aug 2;15:28272. doi: 10.1038/s41598-025-11851-9 (PMC12318116; doi:10.1038/s41598-025-11851-9)
Supplement: Supplementary file 1 — Supplementary Material 1 [file 41598_2025_11851_MOESM1_ESM.docx]

**Table S1. The tentatively identified components from the *Bougainvillea glara* through UPLC/MS^n^**

| **No.** | **Component** | **Molecular Formula** | **Chemical Class** | **R_t_**  **(min.)** | **[M-H]^-^**  ***m/z*** | **[M+H]^+^**  ***m/z*** | **Area %** | **MS/MS fragments** | **Ref.** |
| --- | --- | --- | --- | --- | --- | --- | --- | --- | --- |
| 1 | Caffeic acid hexoside^*^ | C_15_H_18_O_9_ | Phenolic acid | 0.82 | 341 | - | 0.29 | 131 | [1,2] |
| 2 | Luteolin robinobioside^*^ | C_27_H_30_O_15_ | Flavonoid | 6.17 | 593 | - | 2.38 | 501, 372, 285, 236 | [1] |
| 3 | Rosmarinic acid hexoside |  | Phenolic acid | 6.45 | 521 | - | **3.52** | 477, 461, 447, 301, 287, 174 | [3-5] |
| 4 | Rhamnocitrin-*O*-rutinoside | ----- | Flavonoid | 6.62 | - | 609 | 13.60 | 579, 525, 463, 393, 371, 311, 281 | [6] |
| 5 | Sagerinic acid | C_36_H_32_O_16_ | Miscellaneous | 7.11 | 719 | - | **25.03** | 493, 461, 359, 313 | [7] |
| 6 | Apigenin-*C*-hexoside-*C*-pentoside | C_26_H_28_O_14_ | Flavonoid | 7.59 | 563 | - | 1.80 | 501, 395, 359, 343, 303, 191 | [8,9] |
| 7 | Isorhamnetin^*^ | C_16_H_12_O_7_ | Flavonoid | 8.05 | 315 | - | 0.99 | 300, 176, 65 | [1] |
| 8 | Brevifolin-*di*-carboxylic acid hexoside | ----- | Flavonoid | 9.02 | 497 | - | **3.06** | 461, 437, 327, 299, 269, 183 | [10] |
| 9 | Unidentified | ----- | ----- | 10.35 | - | 337 | 7.65 | 315, 301, 283, 242, 214 | ----- |
| 10 | Acanthoside B [(+)-Syringaresinol hexoside]^*^ | C_28_H_36_O_13_ | Lignin | 11.96 | 579 | - | 2.10 | 521, 485, 475, 431, 389, 379, 343, 315, 299, 283 | [11] |
| 11 | Rhamnosyl sophoroside^*^ | ----- | Disaccharide | 12.44 | 487 | - | 2.55 | 445, 427, 359, 345, 329, 301, 279, 179 | [1] |
| 12 | Rosmarinic acid | C_18_H_16_O_8_ | Phenolic acid | 13.56 | 359 | - | 1.82 | 343, 329, 295, 248, 217 | [7,9] |
| 13 | *tri*-*O*-Caffeoyl shikimic acid | ----- | Phenolic acid | 14.22 | 659 | - | **31.39** | 571, 475, 397, 329, 299, 285 | [12] |
| 14 | Chlorogenic acid | C_16_H_18_O_9_ | Phenolic acid | 14.25 | - | 353 | **25.00** | 331, 285, 317, 203 | [4] |
| 15 | Delphinidin hexosyl pentosyl malonate | ----- | Anthocyanidin | 14.86 | - | 683 | 1.10 | 650, 562, 524, 369, 353, 331, 303 | [13,14] |
| 16 | 3-Methyl-epigallocatechin gallate | C_23_H_20_O_11_ | Tannin | 15.70 | 471 | - | 2.14 | 461, 412, 359, 345, 333, 311, 309, 301, 265, 197 | [15] |
| 17 | *O*-[hexosyl-caffeoyl] catalpol (Speedoside) | C_30_H_38_O_18_ | Secoiridoid | 16.20 | 685 | - | **6.61** | 555, 540, 471, 359, 353, 331, 309, 265 | [4,5,16,17] |
| 18 | *di*-Hydro-*bi*-apigenin methyl ether | ----- | Biflavonoid | 16.38 | 553 | - | 1.94 | 535, 471, 441, 353, 331, 311, 209 | [18] |
| 19 | *tri*-Hydroxy-*di*-hydrocyclo-*penta*[b]chromene-dione-carboxylic acid hexoside | ----- | Chromene | 18.84 | 453 | 455 | 1.79 | 447, 441, 397, 361, 337, 325, 289, 265, 191 | [19] |
| 20 | Oleanolic acid | C_30_H_48_O_3_ | Triterpene | 19.08 | 455 | - | 1.86 | 453, 441, 359, 341, 337, 329, 299, 293, 188, 179 | [20,21] |
| 21 | *di*-Hydro-Heveaflavone | ----- | Flavonoid | 21.24 | 581 | 583 | 1.80 | 565, 525, 481, 462, 453, 394, 356, 329, 261, 245, 179 | [18] |
| 22 | Unidentified | ----- | ----- | 27.62 | 815 | - | 0.02 | 787, 721, 695, 669, 655, 627, 481, 331, 265, 189 | ----- |
| 23 | Manniflavanone | ---- | Biflavonoid | 31. 10 | 589 | 605 | 2.50 | 521, 453, 443, 385, 317, 249, 181 | [22] |
|  | | | | | | | | | |
|  | **% identification**  **-ve mode**  **+ve mode** | | | | **93.57%**  **39.70%** | | |  |  |

***for compounds reported before from the same genus.**

1. El-Banna, A.A.; Ghareeb, D.A.; Abdulmalek, S.A.; Abdelrahim, F.A.; Darwish, R.S.; Dawood, H.M. Chemometric-enhanced metabolic profiling of different plant parts belonging to two Bougainvillea species using UPLC-MS/MS spectrometry: Correlation to in vitro anti-inflammatory activity. *Industrial Crops and Products* **2023**, *206*, 117726.

2. El-Tabakh, M.A.; Elhawary, E.A.; Hwihy, H.M.; Darweesh, K.F.; Shaapan, R.M.; Ghazala, E.A.; Mokhtar, M.M.; Waheeb, H.O.; Emam, D.E.; Bakr, N.A. UPLC/ESI/MS profiling of red algae Galaxaura rugosa extracts and its activity against malaria mosquito vector, Anopheles pharoensis, with reference to Danio rerio and Daphnia magna as bioindicators. *Malaria Journal* **2023**, *22*, 368.

3. Barros, L.; Dueñas, M.; Dias, M.I.; Sousa, M.J.; Santos-Buelga, C.; Ferreira, I.C. Phenolic profiles of cultivated, in vitro cultured and commercial samples of Melissa officinalis L. infusions. *Food chemistry* **2013**, *136*, 1-8.

4. Elhawary, E.; Mostafa, N.; Shehata, A.; Labib, R. Comparative study of selected Rosa varieties’ metabolites through UPLC-ESI-MS/MS, chemometrics and investigation of their insecticidal activity against Culex pipiens L. *Jordan Journal of Pharmaceutical Sciences* **2021**, *14*.

5. Sobeh, M.; ElHawary, E.; Peixoto, H.; Labib, R.M.; Handoussa, H.; Swilam, N.; El-Khatib, A.H.; Sharapov, F.; Mohamed, T.; Krstin, S. Identification of phenolic secondary metabolites from Schotia brachypetala Sond.(Fabaceae) and demonstration of their antioxidant activities in Caenorhabditis elegans. *PeerJ* **2016**, *4*, e2404.

6. ElKhateeb, A.; Hussein, S.; Salem, M.; El Negoumy, S. LC-ESI-MS Analysis, Antitumor and Antiviral activities of Bosica senegalensis aqueous methanolic extract. *Egyptian Journal of Chemistry* **2019**, *62*, 77-83.

7. Gkioni, M.D.; Zeliou, K.; Dimaki, V.D.; Trigas, P.; Lamari, F.N. GC-MS and LC-DAD-MS Phytochemical Profiling for Characterization of Three Native Salvia Taxa from Eastern Mediterranean with Antiglycation Properties. *Molecules* **2022**, *28*, 93.

8. Al-Yousef, H.M.; Hassan, W.H.; Abdelaziz, S.; Amina, M.; Adel, R.; El-Sayed, M.A. UPLC-ESI-MS/MS profile and antioxidant, cytotoxic, antidiabetic, and antiobesity activities of the aqueous extracts of three different Hibiscus Species. *Journal of Chemistry* **2020**, *2020*, 1-17.

9. Marzouk, M.M.; Hussein, S.R.; Elkhateeb, A.; El-Shabrawy, M.; Abdel-Hameed, E.-S.S.; Kawashty, S.A. Comparative study of Mentha species growing wild in Egypt: LC-ESI-MS analysis and chemosystematic significance. *Journal of Applied Pharmaceutical Science* **2018**, *8*, 116-122.

10. Mekam, P.N.; Martini, S.; Nguefack, J.; Tagliazucchi, D.; Stefani, E. Phenolic compounds profile of water and ethanol extracts of Euphorbia hirta L. leaves showing antioxidant and antifungal properties. *South African Journal of Botany* **2019**, *127*, 319-332.

11. Saleem, H.; Htar, T.T.; Naidu, R.; Anwar, S.; Zengin, G.; Locatelli, M.; Ahemad, N. HPLC–PDA polyphenolic quantification, UHPLC–MS secondary metabolite composition, and in vitro enzyme inhibition potential of Bougainvillea glabra. *Plants* **2020**, *9*, 388.

12. Gouveia, S.; Castilho, P.C. Characterisation of phenolic acid derivatives and flavonoids from different morphological parts of Helichrysum obconicum by a RP-HPLC–DAD-(−)–ESI-MSn method. *Food Chemistry* **2011**, *129*, 333-344.

13. El-sayed, M.; Abbas, F.A.; Refaat, S.; El-Shafae, A.M.; Fikry, E. UPLC-ESI-MS/MS Profile of The Ethyl Acetate Fraction of Aerial Parts of Bougainvillea'Scarlett O'Hara'Cultivated in Egypt. *Egyptian Journal of Chemistry* **2021**, *64*, 793-806.

14. Irinmwinuwa, E.O.; Cherechi, N.C.; Oyate, G.B.; Ifeyinwa, O.C.; Chinedu, J.O.; John-Iganga, A.A. A comprehensive review of phytochemistry and antibacterial action of Tectona grandis. *International Journal of Science and Research Archive* **2023**, *9*, 133-143.

15. Bastos, D.H.; Saldanha, L.A.; Catharino, R.R.; Sawaya, A.; Cunha, I.B.; Carvalho, P.O.; Eberlin, M.N. Phenolic antioxidants identified by ESI-MS from yerba maté (Ilex paraguariensis) and green tea (Camelia sinensis) extracts. *Molecules* **2007**, *12*, 423-432.

16. Barreira, J.C.; Dias, M.I.; Živković, J.; Stojković, D.; Soković, M.; Santos-Buelga, C.; Ferreira, I.C. Phenolic profiling of Veronica spp. grown in mountain, urban and sandy soil environments. *Food chemistry* **2014**, *163*, 275-283.

17. Yagi, S.; Zengin, G.; Eldahshan, O.A.; Singab, A.N.B.; Selvi, S.; Cetiz, M.V.; Rodrigues, M.J.; Custodio, L.; Dall’Acqua, S.; Elhawary, E.A. Functional constituents of Colchicum lingulatum Boiss. & Spruner subsp. rigescens K. Perss. extracts and their biological activities with different perspectives. *Food Bioscience* **2024**, 104496.

18. Yao, H.; Chen, B.; Zhang, Y.; Ou, H.; Li, Y.; Li, S.; Shi, P.; Lin, X. Analysis of the total biflavonoids extract from Selaginella doederleinii by HPLC-QTOF-MS and its in vitro and in vivo anticancer effects. *Molecules* **2017**, *22*, 325.

19. Fraternale, D.; Ricci, D.; Verardo, G.; Gorassini, A.; Stocchi, V.; Sestili, P. Activity of Vitis vinifera tendrils extract against phytopathogenic fungi. *Natural product communications* **2015**, *10*, 1934578X1501000661.

20. Grati, W.; Samet, S.; Bouzayani, B.; Ayachi, A.; Treilhou, M.; Téné, N.; Mezghani-Jarraya, R. HESI-MS/MS Analysis of phenolic compounds from Calendula aegyptiaca fruits extracts and evaluation of their antioxidant activities. *Molecules* **2022**, *27*, 2314.

21. Wang, S.; Liu, L.; Wang, L.; Hu, Y.; Zhang, W.; Liu, R. Structural characterization and identification of major constituents in Jitai tablets by high-performance liquid chromatography/diode-array detection coupled with electrospray ionization tandem mass spectrometry. *Molecules* **2012**, *17*, 10470-10493.

22. Reed, K.A. Identification of phenolic compounds from peanut skin using HPLC-MSn. Virginia Tech, 2009.
